# Design of a Randomized, Placebo-Controlled, Phase 3 Trial of Tofersen Initiated in Clinically Presymptomatic *SOD1* Variant Carriers: the ATLAS Study

**DOI:** 10.1007/s13311-022-01237-4

**Published:** 2022-05-18

**Authors:** Michael Benatar, Joanne Wuu, Peter M. Andersen, Robert C. Bucelli, Jinsy A. Andrews, Markus Otto, Nita A. Farahany, Elizabeth A. Harrington, Weiping Chen, Adele A. Mitchell, Toby Ferguson, Sheena Chew, Liz Gedney, Sue Oakley, Jeong Heo, Sowmya Chary, Laura Fanning, Danielle Graham, Peng Sun, Yingying Liu, Janice Wong, Stephanie Fradette

**Affiliations:** 1 Department of Neurology, University of Miami, 1120 NW 14th Street, Clinical Research Building, Miami, FL 33136 USA; 2 Department of Clinical Science, Neurosciences, Umeå University, Umeå, Sweden; 3 Washington University School of Medicine, St. Louis, MO USA; 4 The Neurological Institute, Columbia University Irving Medical Center, New York, NY USA; 5 Department of Neurology, Martin Luther University, Halle-Wittenberg, Halle (Saale), Germany; 6 Duke University School of Law, Durham, NC USA; 7 Columbia University Irving Medical Center, New York, NY USA; 8 Biogen, 225 Binney Street, Cambridge, MA 02142 USA

**Keywords:** *SOD1*-ALS, Neurofilament, Genetic testing, *Pre-fALS*, Phenoconversion

## Abstract

Despite extensive research, amyotrophic lateral sclerosis (ALS) remains a progressive and invariably fatal neurodegenerative disease. Limited knowledge of the underlying causes of ALS has made it difficult to target upstream biological mechanisms of disease, and therapeutic interventions are usually administered relatively late in the course of disease. Genetic forms of ALS offer a unique opportunity for therapeutic development, as genetic associations may reveal potential insights into disease etiology. Genetic ALS may also be amenable to investigating earlier intervention given the possibility of identifying clinically presymptomatic, at-risk individuals with causative genetic variants. There is increasing evidence for a presymptomatic phase of ALS, with biomarker data from the Pre-Symptomatic Familial ALS (*Pre-fALS)* study showing that an elevation in blood neurofilament light chain (NfL) precedes phenoconversion to clinically manifest disease. Tofersen is an investigational antisense oligonucleotide designed to reduce synthesis of superoxide dismutase 1 (SOD1) protein through degradation of *SOD1* mRNA. Informed by *Pre-fALS* and the tofersen clinical development program, the ATLAS study (NCT04856982) is designed to evaluate the impact of initiating tofersen in presymptomatic carriers of *SOD1* variants associated with high or complete penetrance and rapid disease progression who also have biomarker evidence of disease activity (elevated plasma NfL). The ATLAS study will investigate whether tofersen can delay the emergence of clinically manifest ALS. To our knowledge, ATLAS is the first interventional trial in presymptomatic ALS and has the potential to yield important insights into the design and conduct of presymptomatic trials, identification, and monitoring of at-risk individuals, and future treatment paradigms in ALS.

**Supplementary Information:**

The online version contains supplementary material available at 10.1007/s13311-022-01237-4.

## Introduction

Despite decades of research, there remains a significant unmet need for effective treatments for amyotrophic lateral sclerosis (ALS). The reasons are manifold, but two key considerations have emerged [1]. First, limited knowledge about the underlying etiology of ALS has made it challenging to select therapeutic strategies that target upstream biological mechanisms of disease. Second, therapeutic intervention often occurs relatively late in the disease course, in part due to the typically long latency from symptom onset to diagnosis [2–4]. However, advances in understanding the genetics of ALS yield potential opportunities to address these challenges. For the subset of patients in whom the genetic cause of ALS is known, therapeutics can be developed to target the underlying genetic defect or a biological mechanism that is proximate in pathophysiology. Moreover, it is possible to identify clinically unaffected individuals with a markedly elevated risk for ALS, and to contemplate early, even presymptomatic, therapeutic intervention with the goal of delaying (or preventing) the appearance of clinically manifest disease, or attenuating disease course following clinical onset [5]. Though precedent in neurodegenerative diseases such as spinal muscular atrophy suggests that intervention prior to significant motor neuron loss could result in improved response to genetically targeted therapies [6], doing so requires an understanding of who to treat and when to treat them, especially since the age at which clinical disease emerges is highly variable in ALS [1, 7].

Approximately 2% of ALS cases are attributed to variants in the superoxide dismutase 1 (*SOD1)* gene (*SOD1*-ALS) [8]. More than 200 *SOD1* variants associated with ALS have been reported [9], with a high degree of variability in penetrance and rapidity of progression [10–14]. Though the mechanisms by which variants in the *SOD1* gene cause degeneration of motor neurons in ALS are not fully understood, these variants are thought to confer a toxic gain of function [8, 15–19]. Tofersen, an antisense oligonucleotide designed to reduce synthesis of SOD1 protein via ribonuclease H-dependent degradation of *SOD1* mRNA [16, 20, 21], is an investigational drug under development for the treatment of *SOD1*-ALS. In clinical studies (NCT02623699, NCT03070119) designed to evaluate tofersen in symptomatic patients with *SOD1*-ALS, intrathecal administration of tofersen 100 mg led to robust reductions in total cerebrospinal fluid (CSF) SOD1 protein, a marker of target engagement, and plasma NfL, a marker of neuronal degeneration [22, 23]. Data from these studies suggest that earlier initiation of tofersen may have a greater impact on preservation of clinical function [22, 23].

Consistent with other neurodegenerative diseases, there is emerging evidence for a presymptomatic phase of ALS — i.e., when the underlying disease process has begun, but clinical manifestations of the disease have not yet appeared [5, 24]. The Pre-Symptomatic Familial ALS (*Pre-fALS)* study, a longitudinal natural history and biomarker study of unaffected individuals at elevated genetic risk for ALS (NCT00317616), was initiated in 2007 with the goals of defining and characterizing the presymptomatic phase of disease, identifying biomarkers of incipient clinical disease, and acquiring the necessary data to facilitate the design of early intervention or disease prevention trials [25]. In the subset of *Pre-fALS* participants with a *SOD1* variant associated with rapid disease progression (e.g., p.Ala5Val [A5V; A4V]) and in whom clinically manifest ALS emerged (i.e., phenoconversion) during follow-up, increased levels of serum neurofilament, most notably neurofilament light chain (NfL), were observed 6–12 months prior to phenoconversion [26, 27]. These data support the potential utility of blood-based measurement of NfL as a biomarker of early disease in SO*D1-*ALS to aid in predicting the timing of phenoconversion [25–27].

The ATLAS study (NCT04856982) is designed to evaluate the impact of initiating tofersen in presymptomatic carriers of *SOD1* variants associated with high or complete penetrance and rapid disease progression who also have biomarker evidence of disease activity (elevated plasma NfL level). Specifically, ATLAS will test the hypothesis that presymptomatic initiation of tofersen will delay the emergence of clinically manifest ALS and/or reduce the loss of function over time as compared to initiation of treatment after emergence of clinically manifest ALS. ATLAS will also expand our understanding of the field of ALS biomarkers and the natural history of disease, particularly its earlier stages.

## Methods

### Overview

**Fig. 1:**
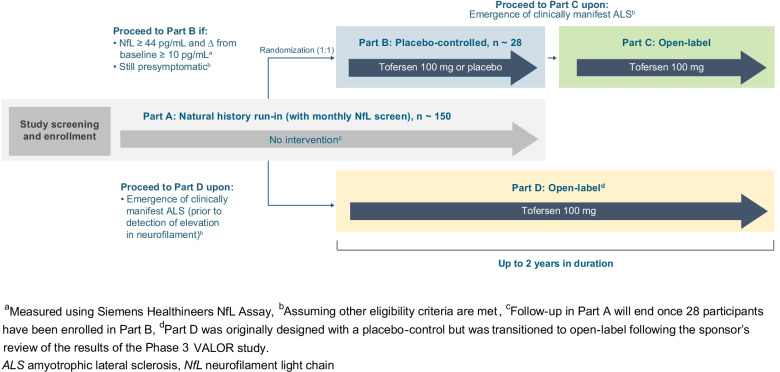
ATLAS study schematic. Study treatment (tofersen or placebo) is administered via intrathecal injection. The dosing regimen consists of three loading doses administered at 14-day intervals followed by maintenance doses once every 28 days thereafter

ATLAS is a global, randomized, placebo-controlled, Phase 3 study with a natural history run-in and open-label extension (Fig. 1). Participants in ATLAS must be at least 18 years of age, carry a *SOD1* variant prespecified in the protocol or approved for inclusion by an independent Mutation Adjudication Committee based on the evidence of high or complete penetrance and association with rapid disease progression, be clinically presymptomatic for ALS, and have a plasma NfL level below the predefined threshold of 44 pg/mL (Siemens Healthineers assay; Malvern, PA, USA).

Part A is a natural history run-in during which participants will be monitored monthly for changes in plasma NfL levels and/or emergence of clinically manifest ALS. Considering feedback from the ALS community, Part A has been designed to minimize burden on study participants, with the opportunity for most assessments to be conducted at home (e.g., monthly blood draws to monitor plasma NfL). If at any point in Part A, a participant is found to have an elevation in plasma NfL level that exceeds the predefined threshold (plasma NfL level ≥ 44 pg/mL *and* an increase of ≥ 10 pg/mL from Part A baseline), no alternative cause unrelated to ALS disease activity for the elevation is identified, and the participant remains clinically presymptomatic, they will have the opportunity to screen for Part B. To reduce potential bias, masked NfL reports will inform only whether a participant has exceeded the predefined threshold, thus ensuring that both participants and investigators remain blinded to specific plasma NfL levels. We anticipate that enrollment in Part A will be completed within ~ 13 months: approximately 15 participants per month for the first 4 months and approximately 10 participants per month for the following 9 months. Enrollment in Part A will determine enrollment in Part B, in which approximately 28 participants will enroll within ~ 4.25 years from study start.

Part B is a randomized, double-blind, and placebo-controlled period in which presymptomatic participants with elevated NfL levels are randomized 1:1 to tofersen or placebo. In Part B, dynamic randomization has been implemented based on *SOD1* variant type (e.g., p.Ala5Val [A5V; A4V] vs. other), last plasma NfL level prior to randomization (< 50 or ≥ 50 pg/mL), and age (< 30 vs. ≥ 30 years). Given the prognostic value of NfL, randomization will be stratified based on whether the last plasma NfL level prior to randomization is above or below 50 pg/mL, the estimated median NfL level at Part B baseline. Moreover, since the *Pre-fALS* participants who developed clinically manifest ALS were largely A5V carriers at least 30 years of age, we elected to stratify randomization on the basis of these factors rather than exclude other variant types or adults (≥ 18 years) under the age of 30. Dynamic randomization helps to ensure that a participant’s treatment assignment depends on their stratification factors, as well as the stratification factors and treatment assignments of previously randomized participants. This approach works to minimize imbalances across all stratification factors (weighted in order of importance as listed above) instead of constructing mutually exclusive strata. Participants who develop clinically manifest ALS in Part B will have the opportunity to receive open-label tofersen in Part C of the study. Together, Parts B and C may last up to ~ 2 years.

Participants who develop clinically manifest ALS prior to Part B randomization may enroll in Part D. Originally designed with a placebo-control (2:1 randomization), Part D was transitioned to open-label following review of the results of the Phase 3 VALOR study (233AS101 Part C). While no other changes to the ATLAS study protocol were deemed necessary, the VALOR data appear to reinforce the importance of early therapeutic intervention as the foundational principle behind ATLAS [23]. A schedule of assessments for study Parts A, B, C, and D can be found in Supplemental Tables 1, 2, 3, and 4, respectively.

**Table 1:** Evaluating the effect of tofersen in presymptomatic *SOD1* variant carriers in ATLAS

**Scientific objectives**	To evaluate whether **presymptomatic** initiation of tofersen (upon elevation of NfL) can:		
	Prevent or delay the emergence of clinically manifest ALS	Reduce the loss of function over time, as compared to delayed tofersen initiation (i.e., upon emergence of clinically manifest ALS)	Reduce the loss of function following emergence of clinically manifest ALS, as compared to delayed tofersen initiation (i.e., upon emergence of clinically manifest ALS)
**PD/efficacy endpoints**	**Primary endpoint**: • Proportion of Part B participants in whom clinically manifest ALS emerges within 12 months of randomization (i.e., Part B baseline) **Secondary endpoints**: • Proportion of Part B participants in whom clinically manifest ALS emerges within 24 months of randomization (i.e., Part B baseline) • Time from Part B baseline to emergence of clinically manifest ALS	**Secondary endpoints**: • Changes from Part B baseline to end of study (~ 24 months) in: o Plasma NfL levels o Total CSF SOD1 protein levels o ALSFRS-R total score o % predicted SVC • From Part B baseline to end of study: o Estimated proportion of deaths or permanent ventilations o Estimated proportion of deaths **Exploratory endpoints:** • Changes from Part B baseline to end of study in: o Home digital assessments o QoL endpoints (i.e., ALSAQ-5, FSS, WPAI, EQ-5D-5L, SF-36)	**Secondary endpoints:** • Changes from Part C baseline to end of study in: o Plasma NfL levels o Total CSF SOD1 protein levels **Exploratory endpoints**: • Changes from Part C baseline to end of study in: o ALSFRS-R total score o % predicted SVC o Home digital assessments o QoL endpoints (i.e., ALSAQ-5, FSS, WPAI, EQ-5D-5L, SF-36) o ROADS o Clinical and Patient Global Impression scales (PGI-S/C, CGI-S/C) • From Part C baseline to end of study: o Estimated proportion of deaths or permanent ventilations o Estimated proportion of deaths
**Analysis population**	Part B population	Combined Parts B and C population	Part C population

*ALSAQ-5* Amyotrophic Lateral Sclerosis Assessment Questionnaire-5, *ALSFRS-R* ALS Functional Rating Scale – Revised, *CGI-C* Clinical Global Impression of Change, *CGI-S* Clinical Global Impression of Severity, *CSF* cerebrospinal fluid, *EQ-5D-5L* EuroQol-5 Dimensions — 5 Levels, *FSS* Fatigue Severity Scale, *NfL* neurofilament light chain, *PGI-C* Patient Global Impression of Change, *PGI-S* Patient Global Impression of Severity, *QoL* quality of life, *ROADS* Rasch Overall ALS Disability Scale, *SF-36* Short Form 36 Health Survey Questionnaire, *SOD1* superoxide dismutase1, *SVC* slow vital capacity, *WPAI* Work Productivity and Activity Impairment Questionnaire

### Endpoints

The ATLAS study was designed to evaluate whether presymptomatic initiation of tofersen (upon elevation of plasma NfL level) can delay the emergence of clinically manifest ALS and/or reduce the loss of function over time as compared with delayed initiation of tofersen (upon emergence of clinically manifest ALS) (Table 1). To evaluate the delay in emergence of clinical onset, the following efficacy measures will be assessed in the Part B population: proportion of participants in whom clinically manifest ALS emerges within 12 months and 24 months of randomization (i.e., Part B baseline) and time from Part B baseline to emergence of clinically manifest ALS. Whether presymptomatic tofersen initiation reduces the loss of function compared with delayed initiation of tofersen will also be assessed by comparing those who receive tofersen in Part B with those who receive tofersen for the first time in Part C. Changes in clinical outcome measures from Part C and Part D baseline to end of study will also be analyzed in Part C and Part D participants, respectively.

For safety endpoints, the incidence of adverse events (AEs) and serious AEs (SAEs) during the treatment period are included (Parts B, C, D). Other exploratory analyses may include pharmacokinetic assessments, biomarkers, digital assessments, and health-related quality of life measures.

### Statistical Analysis

**Fig. 2:**
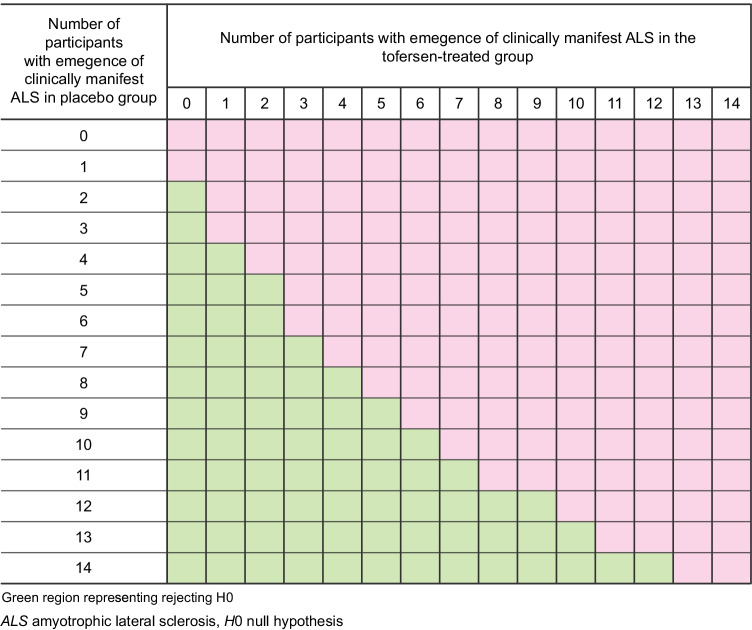
Illustration of decision rules for the primary endpoint

A Bayesian decision rule will be used for the primary efficacy analysis. The number of participants with emergence of clinically manifest ALS by the end of month 12 in the placebo- and tofersen-treated groups is assumed to follow two independent binomial distributions and Jeffreys prior [28]; a noninformative prior of beta (0.5, 0.5) is used. The alternate hypothesis is that the proportion of participants with emergence of clinically manifest ALS by the end of month 12 for the tofersen-treated arm is less than that for the control arm. Based on emergent trial data, the posterior probability that the proportion of participants with emergence of clinically manifest ALS by the end of month 12 for the tofersen-treated arm is less than that for the control arm, will be calculated. If the posterior probability is at least 0.9, the alternative hypothesis is supported. Figure 2 below illustrates the decision rules with the green region representing situations where the alternative hypothesis is supported. The proportion of participants in each arm with emergence of clinically manifest ALS within 24 months of Part B baseline (Day 1) will also be reported, along with corresponding 95% confidence intervals (*CI*s), as a key secondary endpoint. Kaplan–Meier survival curves of the time to emergence of clinically manifest ALS will be presented and used to estimate the median time to event and corresponding 95% *CI*. The change in ALSFRS-R over time from Part B baseline will be summarized descriptively in the combined Part B and Part C dataset. The estimated proportion of participants with the event of death or permanent ventilation and the estimated proportion of deaths since Part B baseline (Day 1) will be reported, along with corresponding 95% *CI*s obtained from Kaplan–Meier analyses based on time to death/permanent ventilation and time to death.

### Neurofilament as a Presymptomatic Biomarker of Disease Activity

**Fig. 3:**
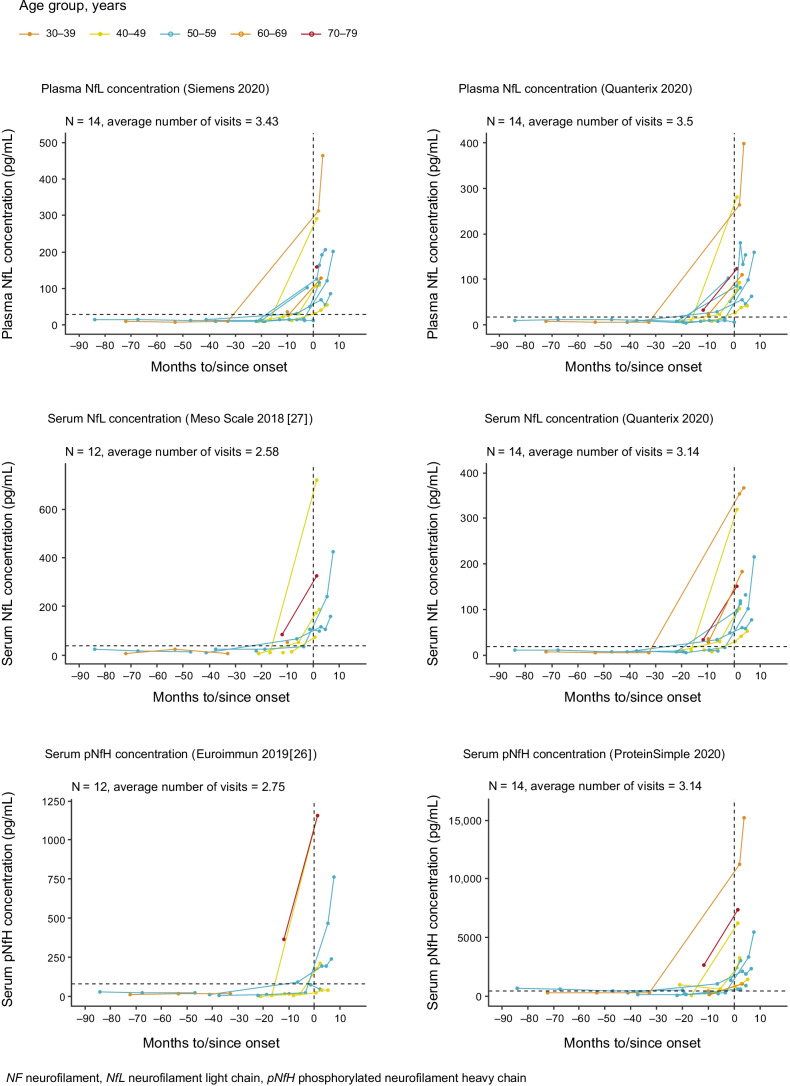
Trends across matrices (serum and plasma), analytes (NfL and pNfH), and platforms (Quanterix Simoa, Siemens Healthineers, ProteinSimple Ella, Euroimmun) of NF prior to phenoconversion. Dotted horizon lines indicate the 95th percentile for the combined control and presymptomatic carrier group. Biological samples and clinical data were from the *Pre-fALS* (Pre-Symptomatic Familial ALS) study

NfL and phosphorylated neurofilament heavy chain (pNfH) levels were evaluated in serum and plasma samples collected from *Pre-fALS* participants with *SOD1* variants. Trends across matrices, analytes, and assays were generally similar, illustrating a rise in neurofilament levels prior to phenoconversion (Fig. 3). For the ATLAS study, the plasma NfL assay by Siemens Healthineers was selected as the platform for NfL analysis based on analytical performance and utilization of a fully automated instrument that reduces sample-to-sample variability, avoids plate bias, and permits real-time testing to enable individual study participant-level treatment decisions. The Siemens Healthineers assay is a 2-site sandwich immunoassay using direct chemiluminometric technology.

**Fig. 4:**
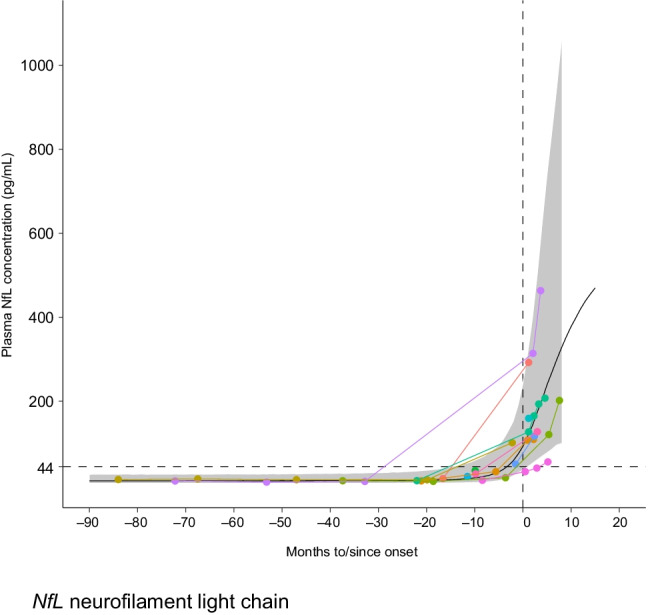
Fitted neurofilament trajectory model (using Siemens Healthineers assay results) to inform the NfL threshold in ATLAS. The black curve represents the fitted model (with 95% prediction bands for the sigmoidal fit shown in gray shading), while the colored lines represent the data. The dotted horizon line indicates the predefined threshold of 44 pg/mL. Biological samples and clinical data were from the *Pre-fALS* (Pre-Symptomatic Familial ALS) study

A trajectory model, modified from the nonlinear *E*_max_ model, was built to predict the time from plasma NfL elevation to phenoconversion using samples from *Pre-fALS* phenoconverters with *SOD1* variants associated with rapid disease progression (Fig. 4). A separate model fitted to the presymptomatic carriers was utilized to generate informative priors for select parameters that quantify NfL level before phenoconversion in the trajectory model for phenoconverters. This fitted trajectory model was used to identify an absolute NfL threshold (44 pg/mL) that minimized the false-positive rate (e.g., the incidence of individuals who met the NfL threshold but did not go on to develop clinically manifest ALS within 12 months) to less than 5% and enabled adequate time for sample processing, screening in Part B, and randomization prior to phenoconversion. Recognizing the potential effect of aging on NfL levels and the absence of an upper age limit for study eligibility, a second criterion of an increase in plasma NfL of at least 10 pg/mL from Part A baseline was introduced. Once a participant’s NfL level meets the dual criteria (≥ 44 pg/mL and ≥ 10 pg/mL increase in NfL from Part A baseline), the investigator will use their clinical judgment to confirm that there are no alternative (non-ALS) causes for the NfL elevation. Based on this NfL trajectory model, the average time from reaching the NfL threshold to emergence of clinically manifest ALS is estimated to be 3–4 months. As such, with ~ 1 month needed for sample processing, screening, and randomization during Part B screening, it is estimated that untreated participants would remain presymptomatic in Part B for an average of ~ 2–3 months before the emergence of clinically manifest ALS in this study.

### *SOD1* Variant Selection

ATLAS is enrolling carriers of gene variants associated with high or complete penetrance and rapidly progressive disease. The selection of this population is largely driven by the fact that, in *Pre-fALS*, the richest phenoconverter data to date are from individuals with rapidly progressive variants (mostly p.Ala5Val [A5V; A4V]). Data from this subgroup were therefore used to model the presymptomatic changes in NfL and to determine the study’s predefined NfL threshold for Part B. This model was then used to estimate the expected phenoconversion rate within 12 months after reaching the predefined NfL threshold and hence inform the ATLAS study’s sample size and duration. The ATLAS study was designed assuming that other variants associated with rapidly progressive disease (beyond p.Ala5Val [A5V; A4V]) would similarly be associated with elevations in NfL level in the 6–12 months prior to the emergence of clinically manifest disease.

Informed by the scientific literature, genetic databases, and clinical experience, a subset of 17 *SOD1* variants was prespecified for inclusion in the protocol: p.Ala5Thr (A4T, A5T), p.Ala5Val (A4V, A5V), p.Cys7Phe (C6F, C7F), p.Cys7Gly (C6G, C7G), p.Asp102Gly (D101G, D102G), p.Asp102His (D101H, D102H), p.Gly115Ala (G114A, G115A), p.Gly42Ser (G41S, G42S), p.Gly86Arg (G85R, G86R), p.Gly86Ser (G85S, G86S), p.Gly94Ala (G93A, G94A), p.His44Arg (H43R, H44R), p.Leu107Phe (L106F, L107F), p.Leu107Val (L106V, L107V), p.Leu39Val (L38V, L39V), p.Arg116Gly (R115G, R116G), and p.Val149Gly (V148G, V149G). Recognizing the evolving understanding of penetrance and disease progression across *SOD1* variants, and the heterogeneity observed across families carrying a given variant, an independent Mutation Adjudication Committee was formed to adjudicate eligibility of *SOD1* variants not specified in the protocol based on available evidence from the participant’s pedigree, scientific literature, and other databases to evaluate the *SOD1* variant criteria of association with high/complete penetrance and rapid disease progression.

### Clinically Manifest ALS

**Fig. 5:**
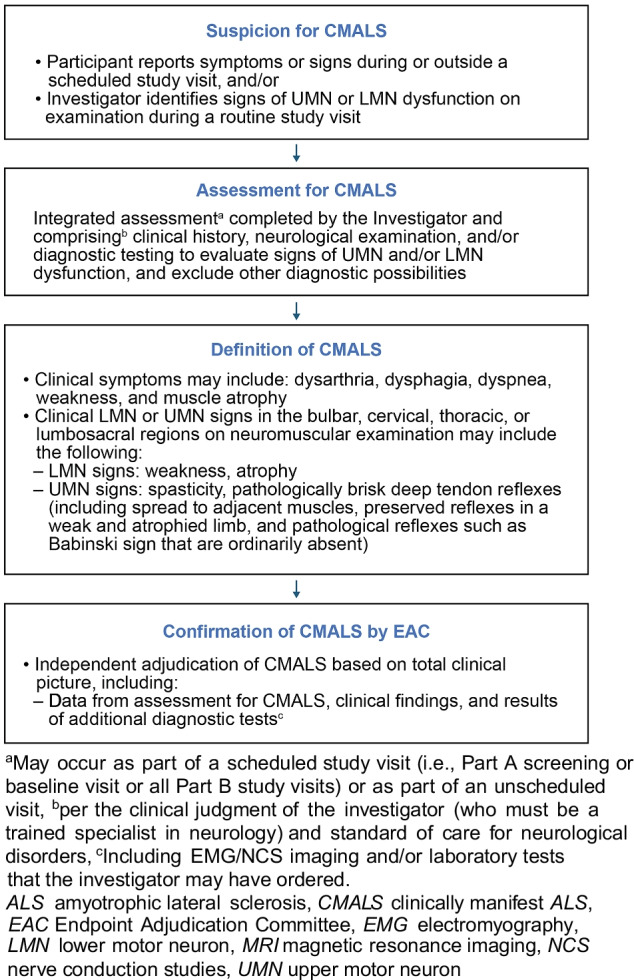
Process for determining the primary endpoint (clinically manifest ALS) in the ATLAS study

ATLAS is designed to evaluate whether initiation of treatment in the presymptomatic stage of disease can delay the onset of clinically manifest ALS. Based on the operational definition of phenoconversion in the *Pre-fALS* study, clinically manifest ALS in ATLAS is defined as the presence of clinical symptoms or signs that, in the context of an integrated clinical assessment completed by a trained neurologist, definitely indicate ALS (Fig. 5) [5, 24]. Suspicion for clinically manifest ALS may be raised if a participant reports symptoms or signs during a scheduled study visit, if a participant reports symptoms or signs outside of a study visit, and/or if the investigator identifies signs of upper motor neuron (UMN) or lower motor neuron (LMN) dysfunction on examination during a study visit in Parts A or B. The participant will then undergo an integrated clinical evaluation performed by the investigator, termed the “Assessment for Clinically Manifest ALS*.*” The Assessment for Clinically Manifest ALS may include clinical history, neurological examination, and diagnostic testing (e.g., electromyography/nerve conduction studies, imaging, and/or laboratory tests), per the investigator’s clinical judgment and standard of care for neurological disorders. The investigator may then determine whether clinically manifest ALS has emerged. Recognizing the potential covert pressure from study participants who may wish to transition from Part B to Part C with the attendant potential bias for declaring that clinically manifest ALS has emerged, we have established an independent Endpoint Adjudication Committee (EAC) to confirm the presence of clinically manifest ALS and establish the timing of its emergence (i.e., phenoconversion). The EAC will blindly review the same source documentation (i.e., clinical history, neurological examination, and results of diagnostic testing) that were available to the investigator and communicate their determination within 12–20 days, permitting timely enrollment into Part C or Part D, as appropriate. Each potential instance of clinically manifest ALS will be independently adjudicated by each of three EAC members, who will be required to reach consensus on whether the case meets the definition of clinically manifest ALS.

The date of emergence of clinically manifest ALS will be defined as the date when the participant first experienced the relevant clinical symptom or, in the absence of symptoms, the date when neurological examination first detected evidence of UMN and/or LMN signs indicative of ALS, whichever is sooner.

### Genetic Testing and Counseling, and Psychosocial Support

In the ATLAS study, participants undergo centralized *SOD1* genetic testing to determine or confirm their *SOD1* variant. Recognizing the complexities of predictive genetic testing and the potential for emotional, psychosocial, legal, and financial implications [25, 29–31], a robust genetic testing and counseling infrastructure has been developed for this study.

During screening for enrollment in Part A of the study, genetic counseling sessions are required before a DNA sample is collected for testing and at the time that the results are communicated. These sessions, which focus primarily on genetic testing, also include in-depth discussions about NfL — specifically, the uncertain but potential implications of above-threshold NfL levels for the risk of imminent clinical disease. Given the potential for continued psychosocial and emotional impact, especially after receiving a positive *SOD1* result and anticipating or learning about a NfL level increase, additional counseling is available as needed throughout the study. Genetic counseling will be performed by qualified individuals as per local standard clinical practice; acknowledging that exact qualifications for providing genetic counseling differs around the world [32]. In addition to these qualifications, genetic counseling providers are required to complete study-specific centralized training. Resources, such as ad hoc consultation with genetic experts, are also available to those at study sites who are providing genetic counseling. For participants and their families, the study website and brochure are available as supplemental materials to help them understand the genetic testing and counseling processes even before enrolling in the study. Moreover, and as with any clinical trial, investigators are responsible for the overall wellbeing of study participants. This includes, but is not limited to, the assessment of psychosocial readiness to enroll in Part A and to undergo genetic and biomarker testing, oversight and monitoring throughout all parts of the study, and support or referral to an appropriate mental health provider as needed based on each study participant’s individual circumstance.

### Ethics and Protections

**Fig. 6:**
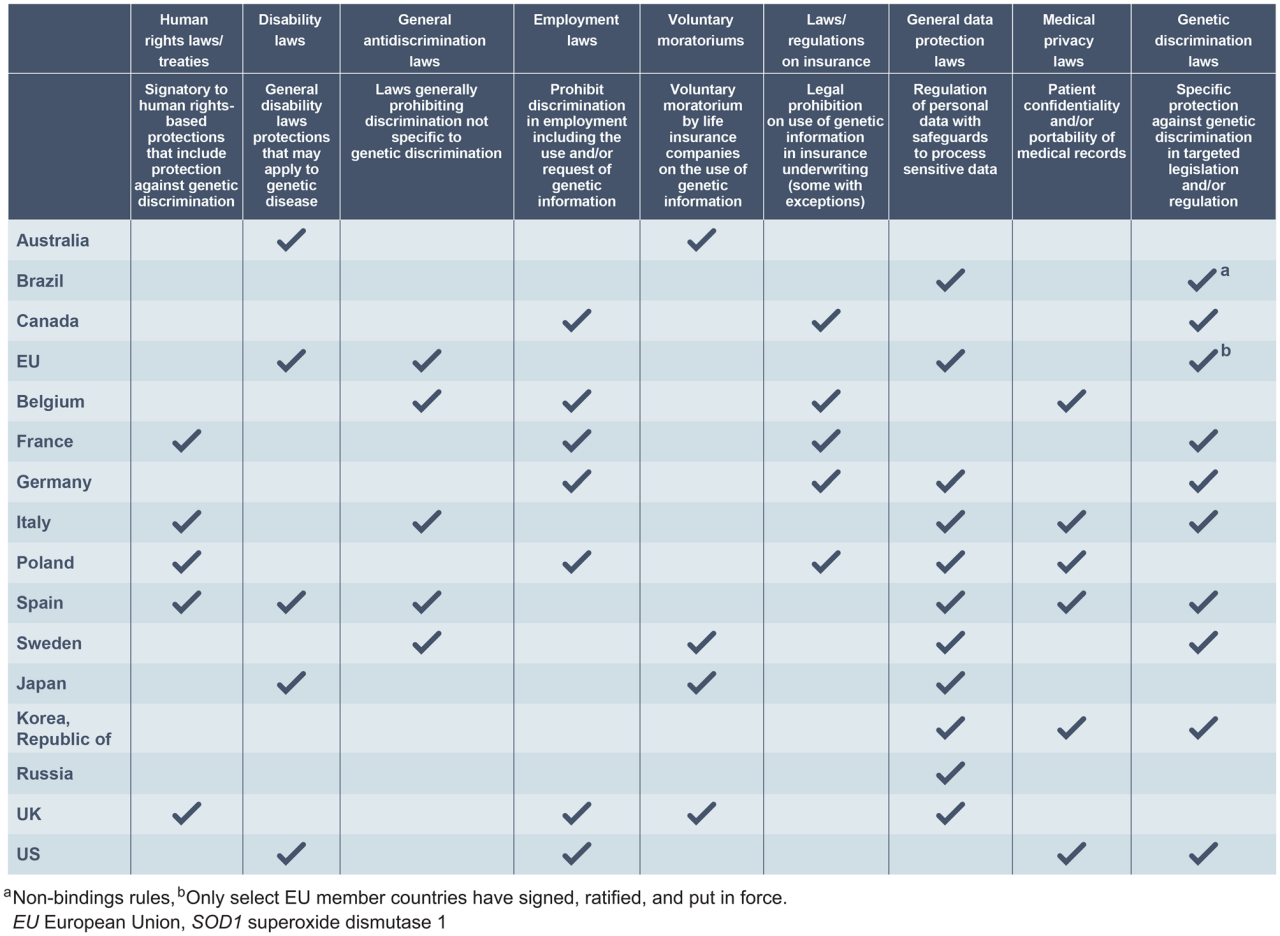
Country-specific privacy and antidiscrimination laws. This figure does not and is not intended to provide a complete or comprehensive description of all country-specific relevant laws, regulations, and practices. This is a starting place to learn the risks and legal protections that individuals undergoing *SOD1* genetic testing may face and is limited to the countries planned to participate in ATLAS

The protection of participants’ privacy — in particular, their *SOD1* variant genetic analysis results and their NfL levels — is paramount. If disclosed, positive *SOD1* test results and elevated NfL levels could be used in discriminatory ways, with potential implications for both the study participant and their biological relatives in many spheres of life, including finances, insurance (disability, long-term, life, etc.), eligibility for educational and recreational activities, family planning, and employment status [30, 33, 34]. For these reasons, ATLAS has implemented multiple approaches to maximize participant privacy. In the USA, a Food and Drug Administration-issued Certificate of Confidentiality provides a legal layer of privacy protection for participants by prohibiting the forced disclosure of identifiable, sensitive research information to anyone not affiliated with the study. US participants also receive protection through the Genetic Information Non-Discrimination Act. Outside the USA, there are numerous protections, including specific genetic nondiscrimination laws, voluntary moratoriums on the use of genetic information by life insurance companies, and broader data protection regimes that offer varying levels of protection to participants (Fig. 6). Study sites are encouraged to keep ATLAS study data, particularly sensitive genetic information and NfL levels, strictly separate from participants’ medical records, and to limit access to data through user-specific authorization where possible. Furthermore, all study sites have been educated about genetic privacy and data management at the ATLAS investigator meetings and reinforced at site initiation visits. Finally, through the informed consent process, potential participants are educated about the benefits, risks, and burdens of presymptomatic genetic testing in addition to how the data will be analyzed, stored, and shared.

## Discussion

The ATLAS study incorporates unique design elements to evaluate whether presymptomatic initiation of tofersen can delay the emergence of clinically manifest ALS and/or reduce the loss of clinical function over time. ATLAS is the first interventional trial in ALS that uses neurofilament as an enrichment marker for study eligibility and a trigger for initiation of study treatment. The design of ATLAS was built upon the premise that elevations in NfL precede the emergence of clinically manifest ALS, as observed in rapidly progressive *SOD1* variant carriers in the *Pre-fALS* study*.* The utility of this critical observation from the *Pre-fALS* study underscores the importance and value of *high-quality* natural history data in advancing therapy development for rare diseases [35].

ATLAS has the potential to aid in the identification and understanding of early markers of disease activity beyond NfL. The collection of CSF (at baseline for Part A and prior to every dose in Parts B/C/D) and urine/blood (annually in Part A; quarterly in Parts B/C/D) will enable the identification of other potential fluid markers of disease activity. Similarly, comprehensive electromyography (EMG) at screening for Part A and Part B may inform the timing of NfL elevation relative to the emergence of EMG abnormalities and the relative sensitivity of these two biomarkers. Through these biomarker assessments, ATLAS will help to inform the conduct of presymptomatic trials in other subsets of the ALS population.

A list of variants associated with high/complete penetrance and rapid disease progression was defined in the protocol based on the totality of information ascertained in the literature, genetic databases, and clinical experience. However, characterization of *SOD1* variants is continuously evolving due to limited reporting and heterogeneity across carriers. To account for this, an independent Mutation Adjudication Committee was formed to adjudicate eligibility of non-protocol-defined *SOD1* variants based on the most up-to-date information at the time. The use of clinically manifest ALS as a primary endpoint is not without challenges, given potential perverse incentive to receive open-label tofersen in Part C; it is for this reason that we have established an independent EAC to confirm the emergence of clinically manifest ALS.

Recognizing the potentially serious and life-long implications of positive genetic testing results on study participants and their families, the study also necessitated the development of a supportive infrastructure to inform, support, and protect prospective participants. Informed by experience from the *Pre-fALS* study, ATLAS aims to take into account two key considerations: (1) when and how participants are informed of their genetic risk, and (2) how participants’ privacy, especially regarding their *SOD1* genetic variant and NfL biomarker status, can be protected. For both study sites and participants alike, education, training, and resources are essential to help address these concerns. Specifically, the ATLAS study has implemented genetic testing and counseling processes along with firewalls and strategies to protect participant privacy in the context of a clinical research study.

## Conclusions

The ATLAS study (NCT04856982) is designed to evaluate the impact of initiating tofersen in presymptomatic carriers of *SOD1* variants associated with high or complete penetrance and rapid disease progression who also have biomarker evidence of disease through elevated plasma NfL. Recognizing that therapeutic intervention in earlier phases of ALS has the potential to yield the greatest clinical benefit, ATLAS, together with data from the VALOR study and its open-label extension study, will inform the optimal timing of treatment initiation in *SOD1*-ALS. As the first interventional trial in presymptomatic ALS, ATLAS has the potential to yield important insights into the design and conduct of presymptomatic trials, identification, and monitoring of at-risk individuals, and future treatment paradigms in ALS and other neurodegenerative diseases.

## Supplementary Information

Below is the link to the electronic supplementary material.

Supplementary file1 (PDF 536 KB)

Supplementary file2 (PDF 589 KB)

Supplementary file3 (PDF 545 KB)

Supplementary file4 (PDF 599 KB)

Supplementary file5 (PDF 535 KB)

Supplementary file6 (PDF 616 KB)

Supplementary file7 (PDF 553 KB)

Supplementary file8 (PDF 580 KB)

Supplementary file9 (PDF 553 KB)

Supplementary file10 (PDF 629 KB)

Supplementary file11 (PDF 563 KB)

Supplementary file12 (PDF 563 KB)

Supplementary file13 (PDF 619 KB)

Supplementary file14 (PDF 598 KB)

Supplementary file15 (PDF 600 KB)

Supplementary file16 (PDF 571 KB)

Supplementary file17 (PDF 545 KB)

Supplementary file18 (PDF 625 KB)

Supplementary file19 (PDF 562 KB)

Supplementary file20 (PDF 527 KB)

Supplementary file21 (PDF 527 KB)

Supplementary file22 (PDF 607 KB)

Supplementary file23 (PDF 329 KB)
