# Correction to: Design of a Randomized, Placebo-Controlled, Phase 3 Trial of Tofersen Initiated in Clinically Presymptomatic *SOD1* Variant Carriers: the ATLAS Study

**DOI:** 10.1007/s13311-022-01286-9

**Published:** 2022-09-29

**Authors:** Michael Benatar, Joanne Wuu, Peter M. Andersen, Robert C. Bucelli, Jinsy A. Andrews, Markus Otto, Nita A. Farahany, Elizabeth A. Harrington, Weiping Chen, Adele A. Mitchell, Toby Ferguson, Sheena Chew, Liz Gedney, Sue Oakley, Jeong Heo, Sowmya Chary, Laura Fanning, Danielle Graham, Peng Sun, Yingying Liu, Janice Wong, Stephanie Fradette

**Affiliations:** 1 Department of Neurology, University of Miami, 1120 NW 14th Street, Clinical Research Building, Room 1318, Miami, FL 33136 USA; 2 Department of Clinical Science, Neurosciences, Umeå University, Umeå, Sweden; 3 Washington University School of Medicine, St. Louis, MO USA; 4 The Neurological Institute, Columbia University Irving Medical Center, New York, NY USA; 5 Department of Neurology, Martin Luther University, Halle-Wittenberg, Halle (Saale), Germany; 6 Duke University School of Law, Durham, NC USA; 7 Columbia University Irving Medical Center, New York, NY USA; 8 Biogen, 225 Binney Street, Cambridge, MA 02142 USA

## Correction to: Neurotherapeutics https://doi.org/10.1007/s13311-022-01237-4

Due to an error during production, captions for Fig. 1, Fig. 5 and Fig. 6 in this article were formatted in a confusing manner in this article as originally published.

The original article has been corrected.

