# Supplementary material for: Design of a Randomized, Placebo-Controlled, Phase 3 Trial of Tofersen Initiated in Clinically Presymptomatic SOD1 Variant Carriers: the ATLAS Study
Source: Neurotherapeutics. 2022 May 18;19(4):1248–58. doi: 10.1007/s13311-022-01237-4 (PMC9587202; doi:10.1007/s13311-022-01237-4)
Supplement: Supplementary file 23 — Supplementary file23 (PDF 329 KB) [file 13311_2022_1237_MOESM23_ESM.pdf]

Supplemental Material

Supplemental Table 1 Schedule of assessments: Part A longitudinal natural history run-in

Assessments	Screening[1]		Longitudinal monitoring[2]		Unscheduled visit for participant report of clinical symptoms or signs of ALS (study site)[3]	Part A ETV/EOS (telemedicine)[4]
	Screening visit 1	Screening visit 2	Baseline (Day 1) [study site]	Day 29 then every 28 days [at home]	As needed	28 days after last visit
**ICF (main)**	✓					
**Future research ICF (optional)**	✓					
**Genetic research ICF (optional)**	✓					
**Part A eligibility criteria**		✓				
**Genetic counseling[5]**	✓	✓ As needed				
**Demographics/medical history[6]**		✓				
**Laboratory assessments**						
Screening tests[7,8]	✓	✓				
CSF samples[8,9]			✓			
NF/Biomarker samples[7,10]	✓		✓	✓		
Blood samples for RNA/DNA			✓			
**Clinical assessments**						
Physical exam/vital signs/EMG[11]		✓	✓			
NfL elevation review[12]				✓		
Assessment for clinically manifest ALS[13]		✓			✓	
Clinical outcome measures[14]			✓			
Digital assessment[15]			✓	✓	✓ Ongoing as needed	
**Participant symptom reporting[16]**	✓ Ongoing as needed					
**Concomitant therapy and procedure reporting[16]**	✓ Ongoing as needed					
**Procedure-related AE/SAE reporting[16]**	✓ Ongoing as needed					

AE adverse event; *ALS* amyotrophic lateral sclerosis; *ALSAQ-5* Amyotrophic Lateral Sclerosis Assessment Questionnaire; *ALSFRS-R* Revised Amyotrophic Lateral Sclerosis Functional Rating Scale; *CSF* cerebrospinal fluid; *EMG* electromyography; *EOS* end of study; *EQ-5D-5L* EuroQoL Group Health Outcome Measure Five-Dimension Five-Level Questionnaire; *ETV* early termination visit; *FSS* Fatigue Severity Scale; *HBV* hepatitis B virus; *HCV* hepatitis C virus; *HIV* human immunodeficiency virus; *ICF* informed consent form; *LP* lumbar puncture; *NCS* nerve conduction study; *NF* neurofilament; *NfL* neurofilament light chain; *SAE* serious adverse event; *SF-36* 36-Item Short Form Health Survey; *SOD1* superoxide dismutase 1; *SVC* slow vital capacity; *WPAI* Work Productivity and Activity Impairment Questionnaire.

[1]Screening visits have been separated into visits 1 and 2 to minimize participant burden; however, following provision of IC, screening assessments can be completed in a single or multiple visits. All screening assessments should be completed within the 42-day screening window

[2]Monitoring continues until participant's plasma NfL level is ≥44 pg/mL and change of ≥10 pg/mL from Part A baseline NfL level, with no alternative identifiable cause, OR until participant has EAC-confirmed clinically manifest ALS, OR until the study completes enrollment of Part B, whichever occurs first. Specific plasma NfL levels will remain blinded to both participants and Investigators

[3]Participant should be evaluated for clinically manifest ALS in the clinic by the Investigator. If clinically manifest ALS is excluded, the participant should remain in Part A of the study. If the Investigator determines that the participant has clinically manifest ALS, the adjudication process will be initiated. If the EAC confirms clinically manifest ALS, the participant can be screened for inclusion in Part D

[4] ETV/EOS visit to be conducted for participants who withdraw consent during Part A (ETV) or who are still being followed in Part A when Part B is fully enrolled (EOS)

[5]Genetic counseling must be conducted before genetic testing is performed, and after genetic testing results are available. Optional genetic counseling is available as needed to participants on an ongoing basis throughout the study

[6]Medical history may be updated throughout Part A as needed

[7]To include blood sampling for *SOD1* variation, HIV, HCV, or HBV detection, NF levels, and clinical laboratory samples for hematology and coagulation at screening

[8]Results of coagulation tests and platelet count must be reviewed before the baseline LP can be performed

[9]LP will be performed to collect CSF samples for biomarker analysis; participants will remain under observation in the clinic for approximately 1 hour after the LP procedure for safety monitoring. Participants will receive a safety follow-up telephone call approximately 24 hours after the procedure

[10]Blood and urine samples for biomarkers will be collected at baseline and then annually (±30 days)

[11]Weight, full physical examination, vital signs (screening and baseline visits); height and comprehensive EMG (screening visit only)

[12]For each instance in which a participant's NfL level is reported to meet the prespecified threshold (≥44 pg/mL and increase of ≥10 pg/mL from baseline), an evaluation of alternative causes of elevated NfL should be performed within 10 days of receipt of NfL report. To evaluate for alternative causes of elevated NfL, the Investigator will contact the participant for evaluation as needed (telephone call/web conference/clinic visit/diagnostic testing). If an alternative cause unrelated to ALS is found, the participant should continue Part A for scheduled visits. If no alternative cause for NfL evaluation is identified, then the participant can proceed to Part B Screening (see Table 2)

[13]Integrated clinical assessment, as per Investigator's clinical judgement, should be performed by an Investigator who is a trained neurology specialist and consists of the following: clinical history, neurological examination (cranial nerves, motor [muscle bulk and tone, function], sensory, reflexes, coordination, and gait), and diagnostic testing (which may include limited/targeted EMG/NCS, imaging and laboratory tests) as clinically appropriate

[14]To include ALSFRS-R, SVC, ALSAQ-5, FSS, WPAI, EQ-5D-5L, SF-36

[15]Digital assessments to be completed at baseline of Part A (in clinic) and every 28 ±7 days thereafter (at home). Digital assessments consist of fine motor/speech tasks and instrumental motor tasks for gait and balance

[16]Any AEs related to study procedures experienced by the participant during Part A from the time of signing the ICF are to be recorded. Concomitant therapy and procedures will be recorded throughout study participation

Supplemental Table 2 Schedule of assessments: Part B randomized double-blind placebo-controlled (presymptomatic phase)

Assessments	Screening visit	Baseline/loading dose visits			Maintenance dose visits			Unscheduled Visit for participant report of clinical symptoms or signs of ALS[2]	ETV/EOS visit for participants not continuing into Part C[3]
	Day −14 to Day −1[1]	Days 1, 15 and 29			Days 57, 85, 113, and every 28 days				28 days after last dose
		Predose	Dose	Postdose	Predose	Dose	Postdose		
**ICF review**	✓								
**Part B eligibility criteria[4]**	✓								
**Genetic counseling[5]**	✓ As needed								
**Randomization**		✓							
**Study drug administration[6]**			✓			✓			
**Medical history**	✓								
**Laboratory assessments**									
Screening tests[7]	✓								
Pregnancy tests[8]	✓	✓			✓				✓
Clinical laboratory samples[6,9,10]	✓	✓			✓				✓
CSF samples[6,11]		✓			✓				
Blood/urine samples[10,12]		✓			✓				✓
**Clinical assessments**									
Physical exam/ECG/EMG/C-SSRS[13]	✓	✓			✓				✓
Vital signs	✓	✓		✓	✓		✓		✓
Assessment for clinically manifest ALS[14]	✓	✓			✓			✓	✓
Clinical outcome measures[10,15]		✓			✓				✓
Digital assessments[16]		✓[10]	✓ Ongoing as needed						
**Concomitant therapy and procedure reporting[17]**	✓ Ongoing as needed								
**Symptom reporting[17]**	✓ Ongoing as needed								
**Procedure-related AE reporting**	✓	✓							
**AE reporting[17]**			✓ Ongoing as needed						
**SAE reporting[17]**	✓ Ongoing as needed								

AE adverse event; *ALS* amyotrophic lateral sclerosis; *ALSAQ-5* Amyotrophic Lateral Sclerosis Assessment Questionnaire; *ALSFRS-R* Revised Amyotrophic Lateral Sclerosis Functional Rating Scale; *CSF* cerebrospinal fluid; *EAC* Endpoint Adjudication Committee; *C-SSRS* Columbia Suicide Severity Rating Scale; *ECG* electrocardiogram; *EMG* electromyography; *EOS* end of study; *EQ-5D-5L* EuroQoL Group Health Outcome Measure Five-Dimension Five-Level Questionnaire; *ETV* early termination visit; *FSS* Fatigue Severity Scale; *HBV* hepatitis B virus; *HCV* hepatitis C virus; *HIV* human immunodeficiency virus; *ICF* informed consent form; *LP* lumbar puncture; *NCS* nerve conduction study; *NF* neurofilament; *NfL* neurofilament light chain; *SAE* serious adverse event; *SF-36* 36-Item Short Form Health Survey; *SVC* slow vital capacity; *WPAI* Work Productivity and Activity Impairment Questionnaire

[1]Day –14 is the day the report that NfL meets prespecified threshold is received at the site

[2]Participants who report clinical symptoms and signs of ALS should be evaluated by the Investigator for clinically manifest ALS. If clinically manifest ALS is not identified the participant should remain in Part B of the study. If clinically manifest ALS is identified the adjudication process is triggered. If the EAC confirms clinically manifest ALS, the participant can be screened for inclusion in Part C (See Table 3). Dosing should continue in Part B until the EAC confirmation and Part C Screening begins. If the EAC does not confirm clinically manifest ALS, the participant should remain in Part B of the study

[3]Participants who do not enroll in Part C or who terminate Part B early will be asked to complete assessments of the ETV/EOS Visit within 28 days after the last dose of study treatment

[4]If a participant develops Investigator-determined and EAC-confirmed clinically manifest ALS prior to randomization in Part B (i.e., during Part B Screening), the participant can enter Screening for Part D. If a participant fails Screening for Part B for another reason, the participant will have an EOS visit in Part B

[5]Optional genetic counseling is also available as needed to participants on an ongoing basis throughout the study

[6]Results of coagulation tests and platelet count must be reviewed before the LP can be performed. Participants will remain under observation in the clinic for approximately 1 hour after the LP procedure for safety monitoring. Participants will receive a safety follow-up telephone call approximately 24 hours after the procedure

[7]To include blood sampling for HIV, HCV, and HBV testing, and FSH testing to confirm postmenopausal status (in postmenopausal female participants only)

[8]To be performed only in women of childbearing potential; results must be negative to continue study participation. Serum test to be performed at Screening and EOS visit; urine or serum test to be performed at all other timepoints. On dosing days, results from urine or serum pregnancy tests must be reviewed predose in order to determine if dose should be administered or held

[9]To include samples for hematology, coagulation, chemistry and urinalysis

[10]May be performed at any time on the day prior to study treatment administration, predose on the day of study treatment administration, or at ETV/EOS visit

[11]LP will be performed to collect CSF samples for PK, PD, safety, and biomarker analysis. Investigator should ensure that the participant does not have contradictions to LP prior to performing LP, as per standard care

[12]To include blood samples for NF, plasma anti-tofersen antibody, PK (for blood samples for NF to PK, to be performed on Days 1 and 29 of Loading Dose and every maintenance visit), and RNA, and blood/urine samples for biomarkers (for blood samples for RNA and blood/urine samples for biomarkers, to be performed on Day 1 only of Loading Dose and every 84-day visit, beginning at Day 85); all blood/urine samples will be performed at ETV/EOS visit

[13]To include weight, full physical/neurological examination (screening, Day 1, and ETV/EOS visit, performance at other timepoints will be per PI's discretion), 12-lead ECG (screening, Day 1, and ETV/EOS visit, performance at other timepoints will be per PI's discretion), comprehensive EMG (screening visit only), C-SSRS (Predose Day 1, every maintenance visit, and ETV/EOS visit only). For C-SSSRS only, may be performed at any time on the day prior to study treatment administration, predose on the day of study treatment administration, or at ETV/EOS visit

[14]Integrated clinical assessment, as per Investigator's clinical judgement, should be performed by an Investigator who is a trained neurology specialist: clinical history, neurological examination (cranial nerves, motor [muscle bulk and tone, function], sensory, reflexes, coordination, and gait), and diagnostic testing (which may include limited/targeted EMG/NCS, imaging and laboratory tests) as clinically appropriate

[15]Includes review of participant self-documented ventilation use, ALSFRS-R (Days 1 and 29 of Loading Dose), SVC, ROADS, ALSAQ-5, FSS, WPAI, EQ-5D-5L, SF-36, PGI-S, CGI-S (for SVC to CGI-S, to be completed on Day 1 of Loading Dose; for SVC to SF-36, to be completed quarterly beginning Day 85), and PGI-C and CGI-C (for PGI-S to CGI-C, performed every 6 months, beginning at Day 169); all clinical outcome measures will be completed at ETV/EOS visit

[16]Digital assessments to be completed predose at Day 1 of Part B (in clinic) and every 28 ±7 days thereafter (at home). Digital assessments consist of fine motor/speech tasks and instrumental motor tasks for gait and balance

[17]Any AEs related to study procedures experienced by the participant during Part A from the time of the signing of the ICF, during Part B prior to the first dose of study treatment, and all AEs experienced by the participants in Parts B, C, or D between the time of the first dose of study treatment and the last follow-up visit are to be recorded. Concomitant therapy and procedures will be recorded throughout participation in the study

Supplemental Table 3 Schedule of assessments: Part C open-label extension

Assessments	Screening visit[1]	Loading dose visits[3]			Maintenance dose visits			ETV or EOS visit
	To be completed ≤28 days after last dose in Part B[2]	Days 1, 15, and 29			Days 57, 85, 113, and every 28 days up to Day 729 (2 years)[4]			28 days after last dose
		Predose	Dose	Postdose	Predose	Dose	Postdose	
**ICF review**	✓							
**Part C eligibility criteria[5]**	✓							
**Genetic counseling[6]**	✓ As needed							
**Study drug administration[7]**			✓			✓		
**Laboratory assessments**								
Clinical laboratory samples[7,8,9]	✓	✓			✓			✓
Pregnancy tests[10]	✓	✓			✓			✓
CSF samples[7,11]		✓			✓			
Blood/urine samples[8,12]		✓			✓			✓
**Clinical assessments**								
Physical exam/ECG/C-SSRS[13]	✓	✓			✓			✓
Vital signs	✓	✓		✓	✓		✓	✓
Clinical outcome measures[8,14]		✓			✓			✓
Digital assessments[15]		✓[8]	✓ Ongoing as needed					
**Concomitant therapy and procedure reporting[16]**	✓ Ongoing as needed							
**AE/SAE reporting[16]**	✓ Ongoing as needed							

AE adverse event; *ALS* amyotrophic lateral sclerosis; *ALSAQ-5* Amyotrophic Lateral Sclerosis Assessment Questionnaire; *ALSFRS-R* Revised Amyotrophic Lateral Sclerosis Functional Rating Scale; *CGI-C/S* Clinical Global Impression of Change/Status; *CSF* cerebrospinal fluid; *C-SSRS* Columbia Suicide Severity Rating Scale; *EAC* Endpoint Adjudication Committee; *ECG* electrocardiogram; *EOS* end of study; *EQ-5D-5L* EuroQoL Group Health Outcome Measure Five-Dimension Five-Level Questionnaire; *ETV* early termination visit; *FSS* Fatigue Severity Scale; *ICF* informed consent form; *LP* lumbar puncture; *NF* neurofilament; *PD* pharmacodynamic; *PGI-C/S* Patient Global Impression of Change/Status; *PK* pharmacokinetic; *ROADS* Rasch Overall ALS Disability Scale; *SAE* serious adverse event; *SF-36* 36-Item Short Form Health Survey; *SVC* slow vital capacity; *WPAI* Work Productivity and Activity Impairment Questionnaire

[1]Screening to be initiated upon EAC confirmation of clinically manifest ALS. If the participant is ineligible for Part C, they will have an EOS visit in Part C

[2]Or ≤14 days of Day 1 or Day 15 loading dose in Part B

[3]For Part C, Loading Dose Period is blinded. Participants randomized to placebo in Part B will receive 3 loading doses of tofersen 100 mg, once every 14 days, and participants randomized to tofersen in Part B will receive tofersen 100 mg on Day 1 and Day 29 and placebo on Day 15 to maintain blinding

[4]Maximum total duration of time spent in Part B and Part C will be approximately 24 months

[5]Includes EAC confirmation of clinically manifest ALS

[6]Optional genetic counseling is available as needed to all participants throughout the study

[7]Results of coagulation tests and platelet count must be reviewed before the LP can be performed. Participants will remain under observation in the clinic for approximately 1 hour after the LP procedure for safety monitoring. Participants will receive a safety follow-up telephone call approximately 24 hours after the procedure

[8]May be performed at any time on the day prior to study treatment administration, predose on the day of study treatment administration, or at ETV/EOS visit

[9]To include samples for hematology, coagulation, chemistry, and urinalysis

[10]To be performed only in women of childbearing potential; results must be negative to continue study participation. Serum test to be performed at screening and ETV/EOS visit; urine or serum test to be performed at all other timepoints. On dosing days, results from urine or serum pregnancy tests must be reviewed predose to determine if dose should be administered or held

[11]LP will be performed to collect CSF samples for PK, PD, safety, and biomarker analysis. Investigator should ensure that the participant does not have contradictions to LP prior to performing LP, as per standard care

[12]To include blood samples for NF, plasma anti-tofersen antibody, PK (for blood samples for NF to PK, to be performed on Days 1 and 29 of Loading Dose and every maintenance visit), and RNA, and blood/urine samples for biomarkers (for blood samples for RNA and blood/urine samples for biomarkers, to be performed on Day 1 only of Loading Dose and every 84-day visit, beginning at Day 85); all blood/urine samples will be performed at ETV/EOS visit

[13]To include weight, full physical/neurological examination (screening, Day 1, and ETV/EOS visit, performance at other timepoints will be per PI's discretion), 12-lead ECG (screening, Day 1, and ETV/EOS visit, performance at other timepoints will be per PI's discretion), C-SSRS (predose Day 1, every maintenance visit, and ETV/EOS visit only). For C-SSSRS only, may be performed at any time on the day prior to study treatment administration, predose on the day of study treatment administration, or at ETV/EOS visit

[14]Includes review of participant self-documented ventilation use, ALSFRS-R (Days 1 and 29 of Loading Dose), SVC, ROADS, ALSAQ-5, FSS, WPAI, EQ-5D-5L, SF-36, PGI-S, CGI-S (for SVC to SF-36, quarterly beginning Day 85, for SVC to CGI-S, Day 1 of loading dose), and PGI-C and CGI-C (for PGI-S to CGI-C, performed every 6 months, beginning at Day 169) ; all clinical outcome measures will be completed at ETV/EOS visit

[15]Digital assessments to be completed predose at Day 1 of Part C (in clinic) and every 28 ±7 days thereafter (at home). Digital assessments consist of fine motor/speech tasks and instrumental motor tasks for gait and balance as a self-administered ALSFRS-R

[16]Any AEs related to study procedures experienced by the participant during Part A from the time of the signing of the ICF, and all AEs experienced by the participants in Parts B, C, or D between the time of the first dose of study treatment and the last follow-up visit are to be recorded. Concomitant therapy and procedures will be recorded throughout participation in the study

Supplemental Table 4 Schedule of assessments: Part D open-label

Assessments	Screening visit[1]	Loading dose visits			Maintenance dose visits			ETV/EOS visit
	Day –28 to Day –1[2]	Days 1, 15 and 29			Days 57, 85, 113, and every 28 days up to Day 729 (2 years)			28 days (±7 days) after last dose
		Predose	Dose	Postdose	Predose	Dose	Postdose	
**ICF review**	✓							
**Part D eligibility criteria[3]**	✓							
**Genetic counseling[4]**	✓ As needed							
**Study drug administration[5]**			✓			✓		
**Medical history**	✓							
**Laboratory assessments**								
Clinical laboratory samples[5-7]	✓	✓			✓			✓
Pregnancy tests[8]	✓	✓			✓			✓
CSF samples[5,9]		✓			✓			
Blood/urine samples[6,10]		✓			✓			✓
**Clinical assessments**								
Physical exam/ECG/C-SSRS[11]	✓	✓			✓			✓
Vital signs	✓	✓		✓	✓		✓	✓
Clinical outcome measures[6,12]		✓			✓			✓
Digital assessments[13]		✓[7]	✓ Ongoing as needed					
**Concomitant therapy and procedure reporting[14]**	✓ Ongoing as needed							
**AE/SAE reporting[14]**	✓ Ongoing as needed							

AE adverse event; *ALS* amyotrophic lateral sclerosis; *ALSAQ-5* Amyotrophic Lateral Sclerosis Assessment Questionnaire; *ALSFRS-R* Revised Amyotrophic Lateral Sclerosis Functional Rating Scale; *CGI-C/S* Clinical Global Impression of Change/Status; *CSF* cerebrospinal fluid; *C-SSRS* Columbia Suicide Severity Rating Scale; *EAC* Endpoint Adjudication Committee; *ECG* electrocardiogram; *EOS* end of study; *EQ-5D-5L* EuroQoL Group Health Outcome Measure Five-Dimension Five-Level Questionnaire; *ETV* early termination visit; *FSH* follicle-stimulating hormone; *FSS* Fatigue Severity Scale; *ICF* informed consent form; *LP* lumbar puncture; *NF* neurofilament; *PD* pharmacodynamics; *PGI-C/S* Patient Global Impression of Change/Status; *PK* pharmacokinetics; *ROADS* Rasch Overall ALS Disability Scale; *SAE* serious adverse event; *SF-36* 36-Item Short Form Health Survey; *SVC* slow vital capacity; *WPAI* Work Productivity and Activity Impairment Questionnaire

[1]If the participant is ineligible for Part D, they will have EOS visit in Part D
[2]Day –28 is the date the site is notified that EAC has confirmed clinically manifest ALS
[3]Includes EAC confirmation of clinically manifest ALS
[4]Optional genetic counseling is available as needed to participants on an ongoing basis throughout the study
[5]Results of coagulation tests and platelet count must be reviewed before the LP can be performed. Participants will remain under observation in the clinic for approximately 1 hour after the LP procedure for safety monitoring. Participants will receive a safety follow-up telephone call approximately 24 hours after the procedure
[6]May be performed at any time on the day prior to study treatment administration, predose on the day of study treatment administration, or at ETV/EOS visit
[7]Includes clinical laboratory samples for hematology, coagulation, chemistry, and urinalysis
[8]To be performed only in women of childbearing potential; results must be negative to continue study participation. Serum test to be performed at screening and EOS visit; urine or serum test to be performed at all other timepoints. On dosing days, results from urine or serum pregnancy tests must be reviewed predose to determine if dose should be administered or held
[9]LP will be performed to collect CSF samples for PK, PD, safety, and biomarker analysis. Investigator should ensure that the participant does not have contradictions to LP prior to performing LP, as per standard care
[10]To include blood samples for NF, plasma anti-tofersen antibody, PK (for NF to PK, to be performed on Days 1 and 29 of Loading Dose and every maintenance visit), and RNA, and blood/urine samples for biomarkers (for RNA and blood/urine samples for biomarkers, to be performed on Day 1 only of Loading Dose and every 84-day visit, beginning at Day 85); all blood/urine samples will be performed at ETV/EOS visit
[11]To include weight, full physical/neurological examination (screening, Day 1, and ETV/EOS visit, performance at other timepoints will be per PI's discretion), 12-lead ECG (screening, Day 1, and ETV/EOS visit, performance at other timepoints will be per PI's discretion), C-SSRS (predose Day 1, every maintenance visit, and ETV/EOS visit only). For C-SSSRS only, may be performed at any time on the day prior to study treatment administration, predose on the day of study treatment administration, or at ETV/EOS visit
[12]Includes review of participant self-documented ventilation use, ALSFRS-R (Days 1 and 29 of Loading Dose), SVC, ROADS, ALSAQ-5, FSS, WPAI, EQ-5D-5L, SF-36, PGI-S, CGI-S (for SVC to CGI-S, to be completed on Day 1 of Loading Dose; for SVC to SF-36, to be completed quarterly beginning Day 85), and PGI-C and CGI-C (for PGI-S to CGI-C, performed every 6 months, beginning at Day 169); all clinical outcome measures will be completed at ETV/EOS visit
[13]Digital assessments to be completed predose at Day 1 of Part D (in clinic) and every 28 ±7 days thereafter (at home). Digital assessments consist of fine motor/speech tasks and instrumental motor tasks for gait and balance as a self-administered ALSFRS-R
[14]Any AEs related to study procedures experienced by the participant during Part A from the time of the signing of the ICF, and all AEs experienced by the participants in Parts B, C, or D between the time of the first dose of study treatment and the last follow-up visit are to be recorded. Concomitant therapy and procedures will be recorded throughout participation in the study
